# Tissue Engineering Bionanocomposites Based on Poly(propylene fumarate)

**DOI:** 10.3390/polym9070260

**Published:** 2017-06-30

**Authors:** Ana M. Diez-Pascual

**Affiliations:** Analytical Chemistry, Physical Chemistry and Chemical Engineering Department, Faculty of Biology, Environmental Sciences and Chemistry, Alcalá University, 28871 Madrid, Spain; am.diez@uah.es; Tel.: +34-918-856-430

**Keywords:** poly(propylene fumarate), graphene oxide, carbon nanotubes, boron nitride nanotubes, biomaterials, mechanical properties, tissue engineering

## Abstract

Poly(propylene fumarate) (PPF) is a linear and unsaturated copolyester based on fumaric acid that has been widely investigated for tissue engineering applications in recent years due to its tailorable mechanical performance, adjustable biodegradability and exceptional biocompatibility. In order to improve its mechanical properties and spread its range of practical applications, novel approaches need to be developed such as the incorporation of fillers or polymer blending. Thus, PPF-based bionanocomposites reinforced with different amounts of single-walled carbon nanotubes (SWCNT), multi-walled carbon nanotubes (MWCNT), graphene oxide nanoribbons (GONR), graphite oxide nanoplatelets (GONP), polyethylene glycol-functionalized graphene oxide (PEG-GO), polyethylene glycol-grafted boron nitride nanotubes (PEG-*g*-BNNTs) and hydroxyapatite (HA) nanoparticles were synthesized via sonication and thermal curing, and their morphology, biodegradability, cytotoxicity, thermal, rheological, mechanical and antibacterial properties were investigated. An increase in the level of hydrophilicity, biodegradation rate, stiffness and strength was found upon increasing nanofiller loading. The nanocomposites retained enough rigidity and strength under physiological conditions to provide effective support for bone tissue formation, showed antibacterial activity against Gram-positive and Gram-negative bacteria, and did not induce toxicity on human dermal fibroblasts. These novel biomaterials demonstrate great potential to be used for bone tissue engineering applications.

## 1. Introduction

**Figure 1:**
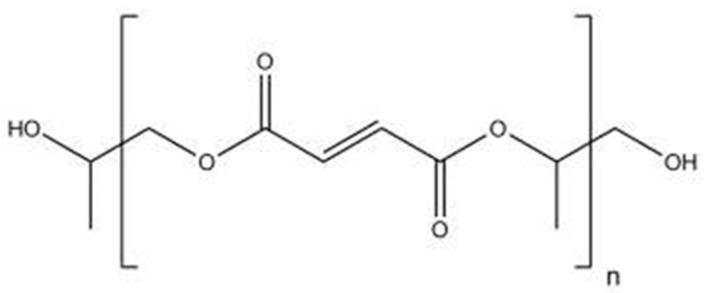
Chemical structure of poly(propylene fumarate) (PPF).

Nowadays, there is a substantial emerging interest for fundamental and applied research on the reinforcement of polymeric materials using nanotechnology. In the biomedical industry, development of novel composite materials with enhanced mechanical properties for tissue engineering applications is of enormous importance. Biodegradable synthetic polymers offer a number of advantages for the development of scaffolds for tissue replacement, including tailorable mechanical properties and degradation kinetics. Furthermore, they can be fabricated into various shapes with desired morphologic features and allow easy incorporation of different chemical groups, facts that favor the growth of tissues [1]. The most frequently employed synthetic biopolymers for tissue engineering are members of the polyester family such as poly(l-lactic acid) (PLLA), poly(glycolic acid) (PGA) and poly(ε-caprolactone) (PCL). Nonetheless, their fragility, poor barrier performance and deficiency of functional diversity in the backbone have limited their applications [1]. Recently, polyhydroxyalkanoates (PHAs), natural polyesters synthesized by bacterial fermentation of sugar or lipids [2] and polyesters based on fumaric acid, a constituent of the Krebs cycle, have attracted a lot of interest for biomedical uses due to their superior biocompatibility and biodegradability [3]. Among them, the most extensively investigated is poly(propylene fumarate) (PPF), a linear polyester incorporating two ester bonds and one unsaturated carbon-carbon double bond (Figure 1) that allows cross-linking either by free radical polymerization with monomers of methyl methacrylate (MMA) or *N*-vinyl pyrrolidinone (NVP) or by means of photoinitiation in the presence of photoinitiators like bisacylphosphine oxide (BAPO) [4]. Cross-linked PPF can suit a number of medical requirements such as biocompatibility, osteoconductivity, sterilizability, and handling characteristics [5]. PPF degrades by simple hydrolysis of the ester bonds and the degradation time depends on polymer characteristics such as molecular weight, type of cross-linkers, and cross-linking density [6]. The degradation products are non-toxic fumaric acid and propylene glycol.

For use in orthopedics, PPF is often combined with particles of ceramic materials such as hydroxyapatite (HA), calcium carbonate, or calcium phosphate [7]. These biocomposites display compressive strengths ranging from 2 to 30 MPa, optimal osteoconductivity and ability to promote osteoblastic cell attachment, hence are suitable for replacement of cancellous bone. In order to further improve the mechanical properties of PPF and extend its range of applications, new approaches are followed such as the addition of nanofillers [8,9,10,11,12,13,14,15], polymer blending [16] or synthesis of copolymers [17,18]. Other methods to additionally improve the mechanical performance of PPF/nanofiller composites are the covalent and non-covalent functionalization of the nanostructures to avoid the formation of aggregates and the reduction of the nanofiller aspect ratio.

To achieve stable nanofiller dispersions and control the microstructure of the nanocomposites, non-covalent or covalent functionalization with polymers may be crucial. The non-covalent approach consists in the physical adsorption or wrapping of polymers to the filler surface via Van der Waals forces, hydrogen bonding, electrostatic or *π*–*π* stacking interactions; its main advantage is that preserves the nanofiller integrity and properties. The covalent method involves the chemical bonding (grafting) of polymer chains to functional groups of the nanofiller surface; it offers numerous possibilities due to the rich surface chemistry of organic nanofillers. However, it frequently generates defects on the nanofiller surface that have detrimental effects on the mechanical and electrical properties [19].

Polyethylene glycol (PEG), a biocompatible and biodegradable polymer extensively used in the preparation of hydrogels for tissue engineering [1], shows great potential to functionalize nanofillers for biomedical applications. It presents outstanding properties, such as solubility in water and in organic solvents, nontoxicity, low protein adhesion and nonimmunogenicity. Furthermore, the end hydroxyl groups of PEG can be easily modified with various functional groups, such as carboxyl, thiol and acrylate, or anchored to other molecules or bioactive agents. Several studies have been reported on the covalent functionalization of organic nanofillers such as graphene (G) and its derivatives (i.e., graphene oxide, GO) [20] as well as inorganic fillers like MoS_2_ with PEG [21]. However, very few works have dealt with PEG-functionalized graphene (or GO) via non-covalent chemistry [22].

The present review deals with the preparation and characterization of PPF-based bionanocomposites incorporating different amounts of single-walled carbon nanotubes (SWCNT), multi-walled carbon nanotubes (MWCNT), graphene oxide nanoribbons (GONR), graphite oxide nanoplatelets (GONP), molybdenum di-sulfite nanoplatelets (MSNPs), PEG-functionalized graphene oxide (PEG-GO), PEG-grafted boron nitride nanotubes (PEG-*g*-BNNTs) and HA nanoparticles with a view to use them for tissue engineering applications. The nanocomposites were prepared via sonication and thermal curing, and their morphology, biodegradability, cytotoxicity, thermal, rheological, mechanical and antibacterial properties have been carefully analyzed through different techniques. The following sections will describe in detail the influence of the nanofiller type and concentration on the different properties of each type of nanocomposite systems. Finally, conclusions and future perspectives will be summarized.

## 2. Poly(propylene fumarate): Synthesis, Properties and Applications

### 2.1. Synthesis

Traditionally, several processes were employed to synthesize PPF via step-growth copolymerization. These methods can be divided into two categories according to the number of steps of the synthesis: one-step method and multistep method. Using the one-step approach, Frazier et al. [23] synthesized PPF from propylene glycol (PG) and fumaric acid using an acidic catalyst. However, high temperatures were required to eliminate excess PG and impurities and to amplify the polymer chain length. Gresser et al. [24] also synthesized PPF by direct esterification of fumaric acid and PG, catalyzed by p-toluene sulfonic acid.

**Figure 2:**
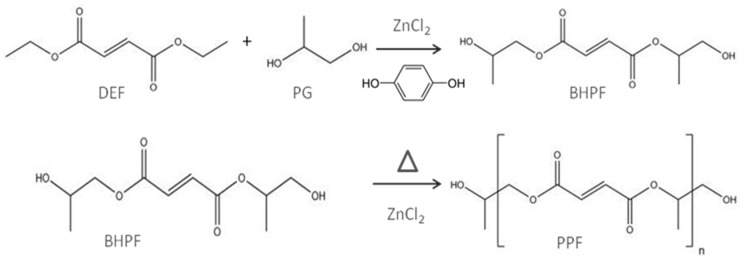
Schematic representation of the two step process used for the preparation of PPF matrix. Taken from [14], with permission from the Royal Society of Chemistry.

Kharas et al. [25] synthesized PPF via a two-step reaction of diethyl fumarate (DEF) and PG. Analogous procedure with optimized experimental conditions was more recently reported in detail by Kasper et al. to synthesize 500–4000 Da PPF [2]. This method has been adapted for the preparation of nanofiller-reinforced PPF composites [10,11,12,13,14,15] (Figure 2). In a first step, both reagents were mixed in the presence of ZnCl_2_ as a catalyst and hydroquinone as a crosslinking inhibitor. This stage resulted in the production of bis(hydroxypropyl) fumarate (BHPF) intermediate and ethanol. In a second step, the intermediate was heated, washed to get rid of the catalyst and lastly dried.

### 2.2. Properties

PPF is an amorphous polymer with a glass transition temperature (T_g_) varying with molecular weight from −30 up to 32 °C. It is a biocompatible and biodegradable copolyester that leads to fumaric acid and PG as main degradation products via hydrolysis of its ester linkages. The PG part in each repeating unit of PPF chain offers one free rotating carbon-carbon single bond. It is an injectable polymer that can be cross-linked in situ through chemical reaction or UV laser. Its mechanical properties are strongly dependent on the molecular weight and degree of crosslinking. Thus, compressive strengths in the range of 2–30 MPa, flexural strengths between 1.8 and 16.1 MPa and flexural moduli ranging between 1.1 and 1.4 GPa have been reported [23,25]. These tailorable mechanical properties make it suitable for developing bone scaffolds. However, it exhibits hydrophobic surface properties that have negative effects on cell adhesion. Copolymerization with hydrophilic polymers such as PEG or modification with peptides is a useful method for increasing PPF hydrophilicity and spreading its range of medical applications [26].

### 2.3. Applications

PPF-based biomaterials have great potential for use in orthopedic tissue engineering owed to their good biocompatibility and controllable mechanical properties. PPF is often combined with ceramic particles like calcium carbonate or calcium phosphate, the resulting materials being appropriate for replacement of cancellous bone [7]. Further, the incorporation of PEG into PPF can reduce platelet adhesion, which is desirable for cardiovascular applications [27]. Besides, copolymers of PPF with polycaprolactone (PCL) have been fabricated as injectable scaffolds for bone defect repair; in particular, they are able to reconstitute the load-bearing capability of vertebral bodies [28]. Bone cements comprising unsaturated PPF and cross-linked PPF microparticles have also been developed for use in craniofacial bone repair applications [29]. On the other hand, block copolymers of PPF and methoxypoly(ethylene glycol) (mPEG) are thermoreversible and suitable for drug delivery [30]. Porous PPF scaffolds could also be useful in various biomedical applications, including magnetic resonance imaging-directed implantation, drug-dispensing materials and/or drug carriers for tumor treatment. In addition, the polymer can be electrospun to yield ultrafine fibers that could be used as biosensors, neural interfaces, drug delivery devices and bioactuators.

## 3. Preparation of Nanofiller-Reinforced PPF Biocomposites

A major challenge in the preparation of PPF-based nanocomposites is to attain a homogeneous dispersion of the nanofillers within the polymer matrix, because the aggregation of the fillers generally reduces the properties of the resulting materials and could limit their applications. In this regard, different strategies including ultrasonication processes, non-covalent and covalent functionalization with polymers have been developed, as will be shown below.

### 3.1. PPF/SWCNT and PPF/HA Nanocomposites

The SWCNTs were synthesized by a high pressure carbon monoxide (HiPco) process, purified and then functionalized by a diazonium-based method [7]. Then, they were dispersed in chloroform and added to a solution of PPF and the cross-linking agent PPFdiacrylate (PPF-DA). Thermal polymerization of the nanocomposites, with SWCNT loadings ≤ 0.2 wt %, was activated by adding benzoyl peroxide (BP) as free-radical initiator and *N*,*N*-dimethyl-*p*-toluidine (DMT) as accelerator; finally the mixture was solution casted and cured. The same method was applied for the preparation of PPF-based nanocomposites with HA loadings in the range of 0–30 wt %. Needle-like HA nanoparticles (20–550 nm in size) were well dispersed within the matrix [15].

### 3.2. PPF/GO Nanocomposites

For the preparation of PPF nanocomposites, GO was non-covalently functionalized with PEG, and disaggregation into thinner layers (c.a. 50 nm size) took place given that the PPF chains intercalated between the GO sheets [13]. The H-bonding interactions between the functional groups of GO and the hydroxyl groups of PEG overcome the *π*–*π* stacking interactions that held the GO flakes together, leading to exfoliation of the nanomaterial, albeit not fully exfoliation was attained since no individual flakes were detected. Further, the histograms of the thickness and width distribution of GO and PEG-GO revealed that the GO distribution of the sheet thickness was unimodal between 20 and 100 nm, with an average value of 65 nm, while the GO width exhibited a wide trimodal distribution in the range of 0.2–1.2 μm, with maximums at 0.25, 0.55 and 0.95 μm, and a mean value of 0.60 μm. In the case of PEG-GO, the polydispersity decreased: both the flake thickness and width distributions were unimodal, with average values of 55 nm and 0.54 μm, respectively. These observations corroborated that the ultrasonication process in the presence of PEG polymer causes the debundling of the GO sheets into thinner layers.

PPF nanocomposites with PEG-modified GO weight percentages in the range of 0.1–3.0 wt % were prepared via sonication and thermal curing [13]. The PEG/GO mixture was suspended in chloroform by sonication and the resulting dispersions were found to be uniform and stable for more than 3 months. Separately, PPF and the cross-linker, NVP, were mixed (1:1 *w*/*w*) and afterward the corresponding amount of the PEG/GOdispersion was added. The mixture was then ultrasonicated, the initiator (BP) was added to begin the polymerization, and the mixture was finally curedunder reduced pressure.

### 3.3. PPF/MWCNT, PPF/GONR, PPF/GONP and PPF/MSNPs Nanocomposites

Firstly, single- and multi-walled graphene oxide nanoribbons (SWGONR and MWGONR, respectively) were synthesized from SWCNTs and MWCNTs via longitudinal unzipping method [12] and subjected to an oxidation treatment. Graphene oxide nanoplatelets (GONP) were synthesized using a modified Hummers method [31] and nano-hexagonal molybdenum disulfite nanoplatelets (MSNPs) were synthesized using ammonium heptamolybdate tetrahydrate, citric acid, and thiourea (CH_4_N_2_S) [32].

AFM images of the SWGONRs revealed that they had a width between 3 and 6 nm and length of 500–1000 nm, confirming that are single layered GO sheets; MWGONRs had a width of 60–90 nm and were 500–1500 nm long. GONPs were disks with a diameter of 10–40 nm and height of 3–5 nm. MSNPs were hexagonal in shape, with a diameter between 50–200 nm and a height of 8 nm.

The preparation of the nanocomposites was similar to that of PPF/GO. The nanomaterials were dispersed in chloroform and then added to the PPF-NVP mixture at concentrations in the range of 0.01 to 0.2 wt %. Thermal cross-linking was carried out using BP and DEF, followed by curing.

### 3.4. PPF/PEG-g-BNNT Nanocomposites

For the preparation of PPF/PEG-*g*-BNNT composites, BNNTs were first synthesized by chemical vapor deposition (CVD) following a variant of the method reported earlier [33]. The synthesized nanotubes displayed typical outer diameters in the range of 30–80 nm and length ≥5 μm. The tubes were uniform and exhibited the distinctive bamboo-like structure decorated with periodically appearing knobs [14].

**Figure 3:**
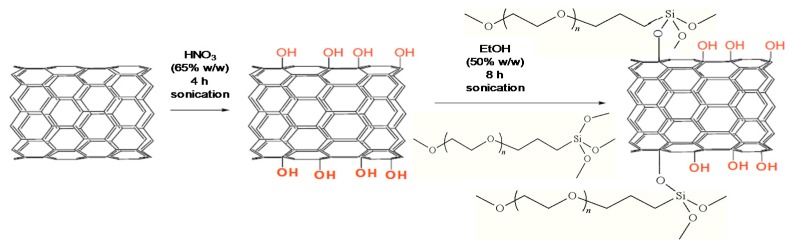
Representation of the covalent functionalization of the BNNTs with PEG. Reprinted from [14], with permission from the Royal Society of Chemistry.

BNNTs were then functionalized by a two stage process, as depicted in Figure 3. Firstly, they were ultrasonicated in a HNO_3_ solution (65% *w*/*w*), leading to hydroxylated nanotubes (BNNTs-OH), with a functionalization degree (FD) of about 13.3% [14]. Secondly, the BNNTs-OH were added to a silane PEG (PEG-Si) solution, and after sonication led to a grafted product (PEG-g-BNNTs). The extent of the grafting reaction was determined as 29%. According to SEM images [14], the PEG-*g*-BNNT is a heterogeneous mixture composed of free PEG segments interacting physically with the BNNT-OH and PEG chains covalently anchored to the BNNT surface. After polymer grafting, the nanotube diameter increased and the bamboo-like arrangement disappeared. The PEG-*g*-BNNT exhibited a cylindrical shape and a rougher surface due to the polymer wrapping. PPF/PEG-*g*-BNNT nanocomposites with nanofiller loadings between 0.1 and 4.0 wt % were also prepared by sonication and thermal curing, similar to PPF/GO, albeit using water instead of chloroform as solvent.

## 4. Characterization of PPF-Based Nanocomposites

### 4.1. Morphology

**Figure 4:**
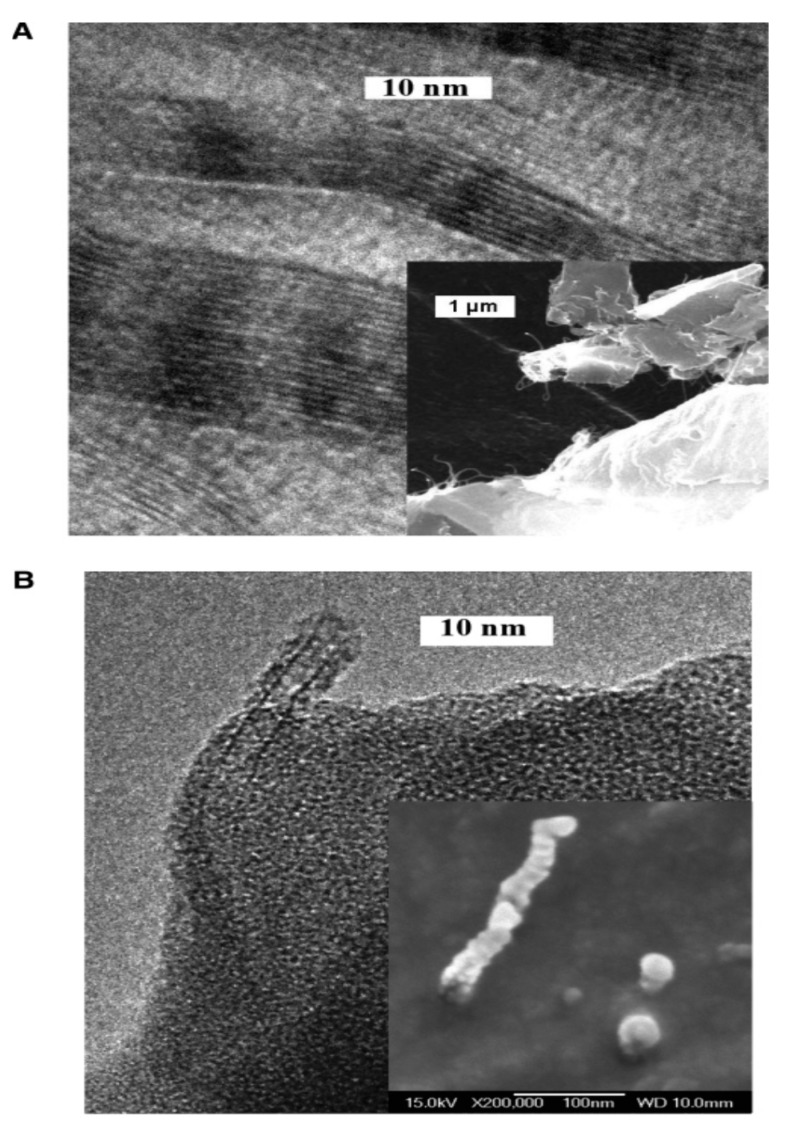
TEM images of PPF nanocomposites with 0.1 wt % SWCNT (**A**) and functionalized SWCNT; (**B**) The lower insets in the images correspond to fractured surfaces of the same samples. Reprinted from [9], with permission from the American Chemical Society.

The morphology of the nanocomposites was investigated by scanning and transmission electron microscopy (SEM and TEM) [9]. At 0.1 wt % loadings, raw SWCNTs formed large nanotube bundles embedded in PPF, and some micron-size aggregates could be observed (Figure 4A). In contrast, the functionalized SWCNTs remained as individual well dispersed tubes or small bundles of only 2–3 nanotubes within the PPF matrix (Figure 4B). This good dispersion was found in all the nanocomposites with functionalized nanotubes. Further, the broken functionalized tubes were covered by a polymer layer, indicating strong nanotube-PPF interactions.

**Figure 5:**
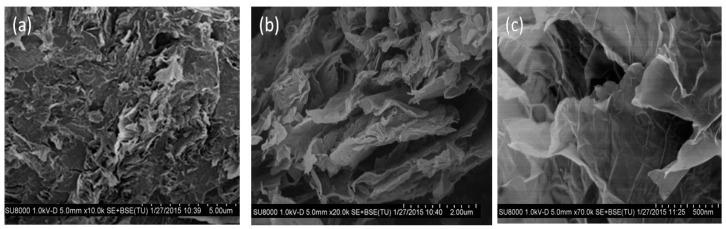
SEM images at different magnifications (**a**) 10.000×, (**b**) 20.000× and (**c**) 70.000× of a PPF/PEG-modified GO nanocomposite with 3.0 wt % GO loading. Adapted from [13], with permission from the American Chemical Society.

Representative SEM images at different magnifications of a PPF/PEG-modified GO nanocomposite with 3.0 wt % GO content are shown in Figure 5. Raw GO powder is composed of stacked nanosheets forming agglomerates via *π*–*π* interactions, van der Waals forces and H-bonding, which disaggregate into thinner layers upon non-covalent functionalization with PEG [13]. These PEG-modify GO flakes are randomly and homogeneously dispersed within the PPF matrix. The micrographs (Figure 5a–c) reveal thin wrinkled GO sheets, with thickness in the range of 7–45 nm and an average value of 18 nm. The sheets are highly disentangled and disaggregated, albeit the lack of individual monolayers corroborates that GO is not fully exfoliated. The images also disclose the crumbled morphology of GO, comprising partially folded thin sheets. As can be observed at higher magnifications (Figure 5c), the sheet edges tend to scroll and fold slightly. Analogous morphology was observed for the other nanocomposites, without the appearance of agglomerates [9].

**Figure 6:**
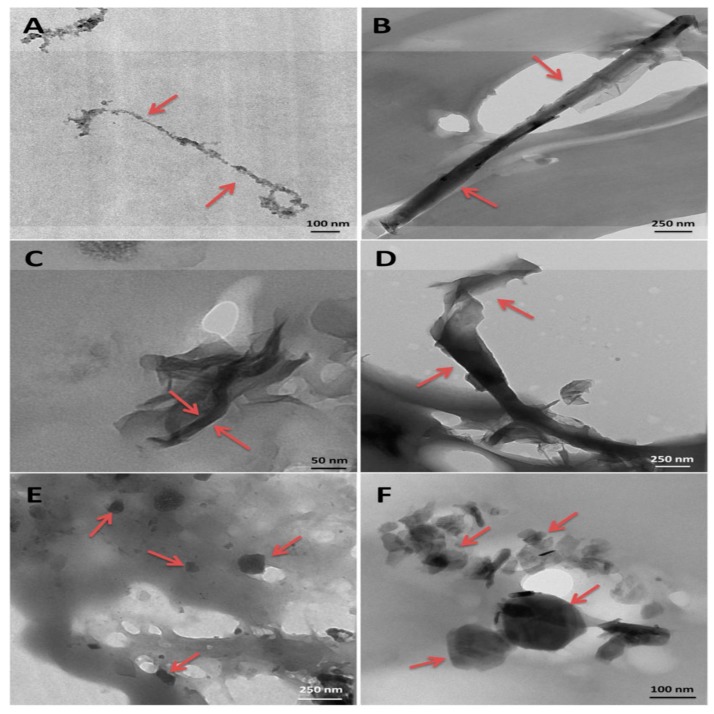
PPF nanocomposites with 0.1 wt % of (**A**) SWCNT; (**B**) MWCNT; (**C**) SWGONR; (**D**) MWGONR; (**E**) GONP and (**F**) MSNP. Reprinted from [12], with permission from the American Chemical Society.

The comparison of TEM images of nanocomposites with 0.1 wt % SWCNTs, MWCNTs, SWGONRs, MWGONRs, GONPs and MSNPs is presented in Figure 6. All the nanomaterials were coated with a thin layer of PPF and embedded within the polymer. SWCNTs and MWCNTs formed bundles of 2–4 and 2–3 nanotubes, respectively; SWGONRs formed bundles of 2–5 nanoribbons, whereas MWGONRs, GONPs and MSNPs existed as individual nanoparticles, which suggests that during the processing the sonication step effectively disrupted the aggregation of nanostructures, thus increasing the surface area available for interaction with the polymer [12].

**Figure 7:**
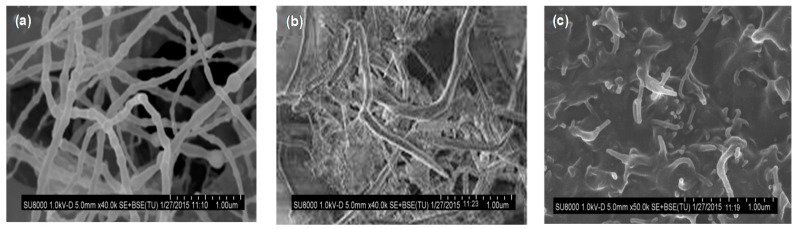
Typical SEM images of BNNTs (**a**), PEG-g-BNNTs (**b**) and PPF/PEG-g-BNNTs (4.0 wt %) (**c**). Adapted from [14], with permission from the Royal Society of Chemistry.

Figure 7 shows the morphology of pristine BNNTs, PEG-*g*-BNNTs and a nanocomposite with 4.0 wt % PEG-*g*-BNNT loading [14]. The neat BNNTs are gathered in small bundles with typical diameters between 30 and 80 nm and length higher than 5 μm (Figure 7a). After the anchoring to the PEG chains, the nanotube diameter somewhat rises, and the bamboo-like structure disappears (Figure 7b). The PEG-grafted nanotubes show a cylindrical shape and a coarser surface, likely due to the polymeric coating on their surface. These grafted nanotubes are well dispersed inside the PPF matrix (Figure 7c), showing a coiled, knotted and bundled structure, and the lack of holes suggests good compatibility between the covalently functionalized tubes and the biopolymer. The polar and H-bonding interactions between the ester groups of PPF and the ether and hydroxyl groups of PEG-*g*-BNNTs hinder nanofiller aggregation and enhance the PPF-BNNTs interfacial adhesion.

### 4.2. Mechanical Properties

Themechanical properties ofbiomaterials are essential for tissue engineering applications; in general, an adequate balance between flexibility and strength is required. The tensile properties of PPF-based nanocomposites, namely the Young’s modulus (*E*) and tensile strength (σ_y_) measured under dry conditions (23 °C and 50% RH) are summarized in Table 1 [13,14]. PPF has an *E* value close to 1 GPa, which increases gradually with increasing nanofiller content, the utmost increase being nearly 200% for the composite with 3.0 wt % PEG-GO and 134% for the nanocomposite with 4.0 wt % PEG-*g*-BNNTs. Interestingly, the reinforcing effect is systematically higher for composites with PEG-GO compared to those incorporating PEG-*g*-BNNTs, despite the reported modulus of the BNNTs (750–1200 GPa) [34] is higher than that of GO (~210 GPa) [35]. The greater improvement for the composites with PEG-GO is possibly related to the more homogenous nanofiller dispersion and stronger nanofiller-PPF interfacial adhesion via hydrogen bonding and polar interactions. Besides, the reinforcement effect found for these GO-reinforced composites is higher than that reported for similar amounts of other nanofillers such as carbon nanotubes [9] or fullerenes [11]. Similar trend is found for the tensile strength (Table 1), being the improvements for composites with PEG-GO considerably larger than those with PEG-*g*-BNNTs. These results suggest that the non-covalent functionalization is more beneficial for improving the mechanical properties of PPF than the covalent one, which could be due to the fact that the oxidation treatment in HNO_3_ acidused forthe grafting of the PEG chains to the BNNTs introduced defects on the nanotube sidewalls that have detrimental effects on the mechanical properties.

The flexural modulus and strength for PPF reinforced with 0.1 and 0.2 wt % SWCNT, MWCNT, SWGONR, MWGONR, GONP and MSNP are compared in Table 1 [12,13,14]. A moderate increase in modulus is found compared to neat PPF. 2D nanostructures led to higher reinforcement than 1D nanofillers, showing increases in the range of 3–40%. In contrast, strong increments are found in the flexural strength, in the range of 50–262%. A clear trend in the reinforcement effect of the nanofillers can be drawn from Table 1: MSNP > GONP > MWGONR > SWGONR > SWCNT > MWCNT. Overall, nanoplatelets are better reinforcing agents than nanoribbons; further, inorganic 2D nanostructures reinforce PPF better than 2D carbon nanostructures.

The values of the Young’s modulus, flexural modulus and flexural strength of PPF-based nanocomposites are lower than those of cortical bone, but higher or comparable to those of trabecular bone, hence seem to be suitable for the replacement of this type of bone tissue. The higher mechanical properties of the 2D nanofiller-reinforced nanocomposites compared to 1D ones may be attributed to different factors, namely surface area, aspect ratio, and crosslinking density [12]. 2D nanoparticles possess higher surface area, which would enable a more effective load transfer from PPF to them. The lower aspect ratio of GONPs compared to GONRs may be responsible for their better mechanical reinforcement. Moreover, the addition of 0.1–0.2 wt % of GONPs, MSNPS and MWGONRs resulted in important increases in the crosslinking density of the nanocomposites, since they have a large number of hydroxyl, carboxyl, and sulfide functional groups on their surface that enable strong non-covalent and covalent interactions with the polymer, thus leading to improved mechanical properties. Similar results were found for thermoset bionanocomposites based on epoxidized vegetable oils: the higher the crosslinking density, the better the mechanical performance [36]. Additionally, these functional groups facilitate a better dispersion of the 2D nanostructures in the polymer matrix and prevent the formation of large aggregates. Another work compared the efficacy of fullerenes and SWCNTs (as synthesized and wrapped with surfactants) as reinforcing agents of PPF [10]. Fullerenes slightly increased the mechanical properties (~10% increase in compressive or flexural modulus). Raw SWCNT led to 65% and 69% increase in the compressive and flexural modulus, respectively, at loadings of 0.02–0.1 wt %. Functionalization of the SWCNTs with surfactants led up to 2-fold increase in the moduli. Among all, MSNPs led to the highest mechanical improvements, suggesting that the chemical composition of the nanostructures may also play a role, and that inorganic 2D nanofillers could be more effective for enhancing PPF performance than carbon nanostructures. However, the addition of 3D HA nanoparticles (10–30 wt %) hardly improved the compressive modulus of PPF [15], ascribed to the formation of large agglomerates as such high nanoparticle loadings.

**Figure 8:**
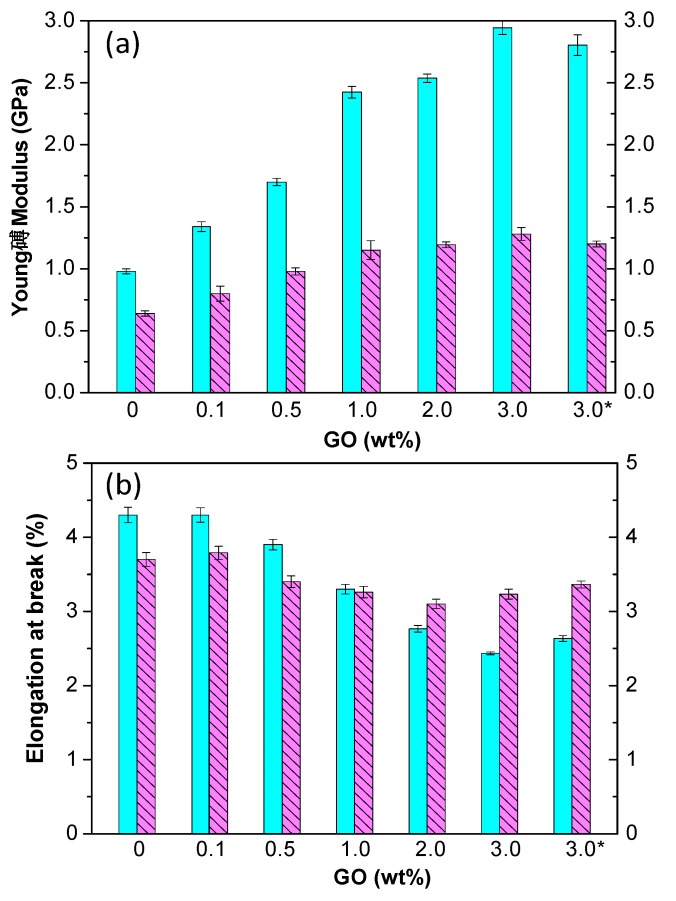
Young’s modulus (**a**) and ductility (**b**) under dry (solid bars) and SBF (dashed bars) conditions for PPF/PEG-GO nanocomposites. Adapted from [13], with permission from the American Chemical Society. The asterisk indicates a composite prepared in the absence of chloroform.

Biomaterials for tissue engineering frequently remain inside the body for a long period of time; therefore, it is important to evaluate the mechanical properties of PPF-based nanocomposites under physiological conditions such as exposure to SBF at 37 °C [13,14] (Figure 8). Systematically, the values of stiffness and strength obtained under dry environment were higher than those in a SBF medium, probably associated to the raise in the level of hydrophilicity and biodegradation rate found upon increasing nanofiller concentration, hence more prominent plasticization effect of absorbed water, which provokes a decrease in the degree of crosslinking between PPF chains. The differences between dry and wet conditions rise with increasing nanofiller loading, since the plasticization effect should be more pronounced owed to the raise in hydrophilicity [13]. The largest differences (56% and 38% in stiffness and strength, respectively) were for the nanocomposite with 3.0 wt % PEG-GO, which incorporates a large number of oxygen-containing groups.

Surprisingly, the elongation at break of PPF and nanocomposites with low nanofiller loading is lower in SBF compared to dry conditions, while that of composites with nanofiller content ≥2.0 wt % is higher (Figure 8) [13,14]. This behavior could be ascribed to the competition of two contradictory factors: the hydrolytic degradation that provokes the breakage of the ester bonds, thus shortening the polymeric chains, and the residual humidity that acts as a plasticizer, thereby increasing the ductilityof PPF. At low nanofiller contents, the first factor prevails, while at loadings ≥2.0 wt % the plasticizing effect predominates, and the consequence is an increase in ε_b_. Nonetheless, the toughness of both types of nanocomposites is systematically lower in a SBF than under dry conditions, indicating that in the nanocomposites with nanofiller concentration ≥2.0 wt % the decrease in tensile strength exceeds the increase in ductility. The largest drops in ductility have been found for nanocomposites with 0.5 and 1.0 wt % PEG-GO (40% and 33%, respectively), whereas the smallest (~17%) for those with 3.0 wt % PEG-GO and 4.0 wt % PEG-*g*-BNNTs [13,14]. The drop in mechanical properties due to immersion in SBF was directly related to the level of hydrophilicity and biodegradation of the nanocomposites: the higher the hydrophilicity and weight loss due to hydrolytic degradation, the larger the loss in mechanical properties. More importantly, PPF-based biocomposites were found to preserve enough mechanical strength under physiological conditions to supply efficient support for new tissue formation.

### 4.3. Thermal Properties

**Figure 9:**
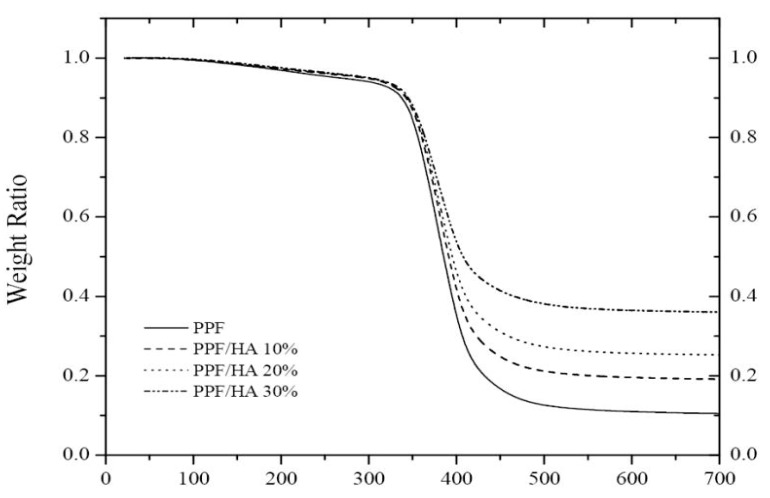
TGA thermograms of cross-linked PPF and the HA nanocomposites. Reprinted from [15], with permission from Elsevier.

The thermal stability of PPF-based nanocomposites has been investigated by thermogravimetric analysis (TGA) under a nitrogen atmosphere [13,14,15]. Cross-linked PPF exhibits a single degradation step (Figure 9) that starts at 285 °C and exhibits the maximum rate of weight loss at 342 °C. This decomposition temperature increases with increasing HA loading, by about 20 °C at 30 wt % HA.

Further, the residue strongly increases upon addition of the nanoparticles, pointing towards improved flammability behavior of the nanocomposites compared to the neat biopolymer. Stronger increments were reported for PPF/PEG-GO nanocomposites, by up to 43 °C at 3.0 wt % loading [13], likely because the GO nanosheets can act as efficient barriers and hinder the transport of volatile products from the bulk of the matrix to the gas phase.

### 4.4. Rheological Properties

**Figure 10:**
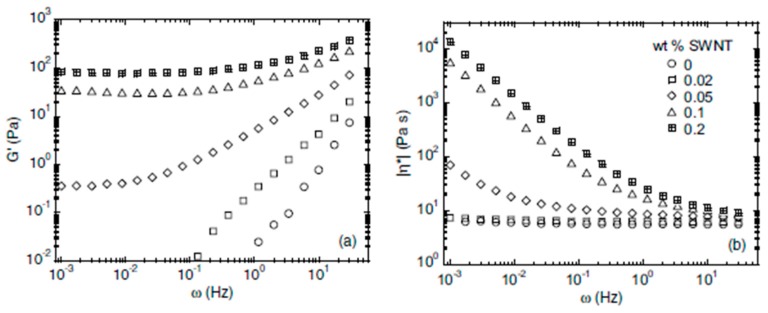
Frequency (ω) dependence of the elastic modulus (**a**) and complex viscosity (**b**) for PPF and the SWCNT reinforced composites. Adapted from [10], with permission from IOP Publishing.

Dynamic oscillatory shear measurements have been carried out to investigate the rheological properties of PPF and nanocomposites with different SWCNT content (Figure 10). Both the elastic modulus (*G*´) and the complex viscosity magnitude (η) change suddenly from liquid-like behavior for the polymer matrix to a solid-like behavior for the nanocomposites with 0.05 wt % SWCNT and higher [10]. At low frequencies, the values of *G*’ increase almost linearly with increasing SWCNT concentration, which demonstrates that the SWCNTs effectively act as reinforcements. Based on the linear extrapolations of these low frequency plateau values of *G*’, a percolation threshold of ~0.03 wt % was calculated [10]. This dependence suggests an effective aspect ratio for the SWCNTs in the range of 2000–3000. Overall, rheological measurements confirmed the good dispersion of the SWCNTs up to 0.05 wt %. At higher loadings, aggregation occurred due to the strong increase in the nanocomposite viscosity that prevented efficient diffusion of the nanotubes within the polymer.

### 4.5. Antibacterial Properties

**Figure 11:**
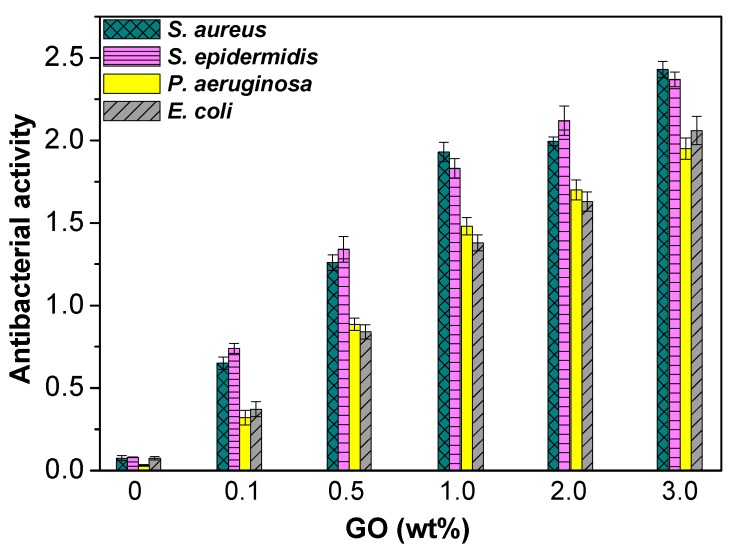
Antibacterial activity of PPF/PEG-GO nanocomposites. Adapted from [13], with permission from the American Chemical Society.

Microbial infection of biomaterials is a widespread issue in surgical treatment because it may lead to implant release, arthrodesis and even death. Among the most common bacteria involved in the contamination of biomaterials are Gram-negative *E*. *coli* and *P*. *aeruginosa* as well as Gram-positive *S*. *aureus* and *S*. *epidermidis* [37]. Metal nanoparticles (Ag, Cu, Au), metal oxide nanomaterials (TiO_2_, ZnO, MgO), and carbon nanotubes are the most frequently employed nanofillers to develop antimicrobial action [38]. Thus, the antibacterial characteristics of PPF-based nanocomposites have been explored against the indicated bacteria [13,14]; the nanocomposites were first sterilized and then immersed in a nutrient broth of ~2.0 × 10^6^ colony forming units per mL (CFU/ mL). Subsequent to incubation at 37 °C for 1 day, the number of viable bacteria colonies was counted. The antibacterial activity was calculated as: log(viable cell count_control_/viable cell count_composite_), where a glass without sample was employed as control. Results obtained for PPF/PEG-GO nanocomposites are shown in Figure 11. Analogous trend was found for composites reinforced with PEG-*g*-BNNTs [14].

According to the ISO 22196:2007 standard, efficient antibacterial activity should be higher than 2. Pure PPF does not exhibit antimicrobial action versus any of the bacteria investigated. For both types of composites, the antibacterial action increased with increasing nanofiller content, and efficient antibacterial activity was only attained for 2.0 wt % PEG-GO against *S*. *aureus* and *S*. *epidermidis*, 3.0 wt % PEG-GO against all the bacteria and 4.0 wt % PEG-*g*-BNNTs against *E*. *coli* and *P*. *aeruginosa*. Further, compositesreinforced with PEG-GO displayed better activity than the PEG-*g*-BNNT counterparts. This stronger bacterial inactivation could be rationalized considering the very large GO surface area that leads to a very big GO-bacteria contact area. Interestingly, in the nanocomposites with PEG-GO the biocide effect was stronger versus Gram-positive cells [13], whereas for those with PEG-*g*-BNNTs the antibacterial activity is higher against the Gram-negative ones [14]. This points towards different antibacterial mechanisms albeit has not been elucidated yet. Besides, very small differences are detected between the activity towards *S*. *aureus* and *S*. *epidermidis*, and the same happens for the inactivation of *E*. *coli* and *P*. *aeruginosa*, suggesting that the different toxicity is principally associated to the different nature of the cell wall between Gram-negative and Gram-positive bacteria [39]. Moreover, the differences could be connected to their dissimilar shape and size: *S*. *aureus* and *S*. *epidermidis* are tiny rounded bacterium, while *E*. *coli* is a short rod-shaped and *P*. *aureginosa* a very long rod-shaped microorganism.

**Figure 12:**
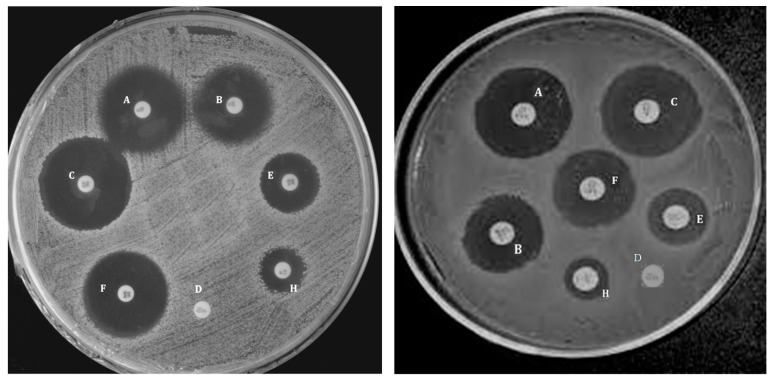
Inhibition zone of PPF/PEG-g-BNNTs nanocomposites on *E*. *coli* (**left**) and *S*. *aureus* (**right**) measured via the agar-diffusion technique. The wells corresponding to each nanocomposite loading are the following: (**A**) 4.0 wt %; (**B**) 1.0 wt %; (**C**) 0 wt % (positive control); (**D**) 0 wt % (PPF); (**E**) 0.5 wt %; (**F**) 2.0 wt %; (**H**) 0.1 wt %. Reprinted from [14], with permission from the Royal Society of Chemistry.

Additional information about the antibacterial action of these nanocomposites was obtained via the agar-diffusion technique, which establishes the sensitivity of microorganisms towards specific antimicrobial agents; the bigger the zone of inhibition, the more susceptible the bacteria are. For such experiments, each bacterium was grown during the night in a nutrient agar, and wells of 6 mm diameterwere bored in the medium with the help of a glass borer; subsequently, 50 µL of the nanocomposites were loaded into each well, and the inhibition zone was measured after incubation at 37 °C for 24 h. Figure 12 presents the pictures of the inhibition zone versus *E*. *coli* and *S*. *aureus* for PPF/PEG-*g*-BNNT composites [14]. Cefixime and cefoperazone, which are third generation antibiotics, were employed as blanks against *E*. *coli* and *S*. *aureus*, respectively. It can be observed that these nanocomposites have stronger antibacterial activity against the Gram-negative bacteria, and that the distinct activity versus the two types of bacteria diminishes upon increasing nanofiller concentration, consistent with the results obtained from the colony-counting technique. PPF does not displayinhibition zone against any of the bacterium, demonstrating thatthe microorganisms tested are resistant to this copolymer. Thediameters of the inhibition zone increase as the nanofiller loading rises, and the nanocomposite with 4.0 wt % displays a zone of inhibition of 19 mm, similar to that of cefoperazone antibiotic against *S. aureus*, and only a little smaller than that of cefixime versus *E*. *coli*, which is about 22 mm. These facts corroborate the vulnerability of the bacteria to this type of nanocomposites, which can be efficiently used as antimicrobial agents.

The antibacterial activity of graphene-based materials is well documented [40], although the reasons for their biocide action are not fully understood so far. A number of mechanisms have been described like oxidative stress due to the generation of reactive oxygen species (ROS), cell membrane damage provoked by direct contact of the bacteria with the edges of the GO nanowalls or entrap of microorganisms within the graphene nanosheets [41]. In particular, a few works have supplied confirmation of the ROS generation of GO on bacterial systems [42]. The GO nanosheets can produce hydroxyl radicals that attack the CO moieties of the peptide bonds of the bacterial cell wall and harm the cellular components, consequently destroying the bacteria. Thus, the Gram-negative bacteria comprising an outer membrane are more resistant to the membrane damage induced by the GO flakes than the Gram-positive ones that do not have an outer membrane.

On the other hand, very little literature about the antibacterial action of materials incorporating BNNTs has been reported, and likely mechanisms could also be the formation of ROS, the capacity to carry out endocytosis and membrane breakage induced via introduction of the BNNTs in the cell membrane [43]. Indeed, BNNTs have been found to be spontaneously attracted by lipid bilayers and are able to penetrate cell membranes via a lipid-mediated inclusion mechanismanalogous to that described for CNTs [44]. Consequently, the lipidmembrane disruption may be considered as the foremost reason for the antibacterial action of nanocomposites filled with BNNTs, hence the Gram-negative comprising a peptidoglycan layer are less resistant to the damage caused by these inorganic nanotubes.

### 4.6. Cytotoxicity and Biodegradation

**Figure 13:**
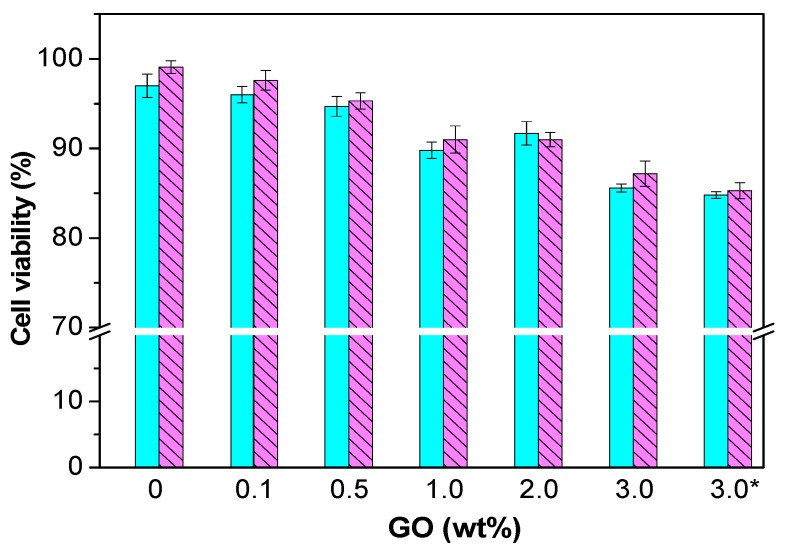
Cell viability of NHDF cultured in the presence of PPF/PEG-GO nanocomposites after 24 h (solid lines) and 72 h (dashed lines). Reprinted from [13], with permission from the American Chemical Society.

Prior to use these nanocomposites for biomedical applications, in vitro cytocompatibility studies have to be carried out. Normal human dermal fibroblasts (NHDF) are one of the most frequent cells that can interact with bone tissue scaffolds, hence they have been chosen to assess the cytotoxicity of PPF and different nanocomposites. Results obtained for PPF/PEG-GO nanocomposites are shown in Figure 13. Pure PPF, a fully biodegradable biopolymer, does not show any toxicity to NHDF, showing a cell viability of about 99%, in agreement with preceding works that showed the superior biocompatibility of PPF with diverse cells [4]. All the PPF/PEG-GO and PPF/PEG-*g*-BNNTs nanocomposites can be considered as non-toxic, given that exhibit cell viability values >85% [13,14]. However, the viability diminishes slightly upon raising nanofiller loading, the largest falls being about 14% and 11% for nanocomposites with 3.0 wt % PEG-GO and 4.0 wt % PEG-*g*-BNNTs compared to PPF.

A few groups have investigated the in vitrocytotoxic effects of GO and BNNTs, and contradictory results have been reported [43,45]. These contradictions are ascribed to the large number of factors influencing the toxicity of nanomaterials like concentration, size, presence of defects, synthesis procedure and surface modification as well as the cell type. Some works pointed that these nanomaterials can induce cell damage via different mechanisms, the most important being induction of oxidative stress and DNA damage that can result in cell death [43,44]. It is generally accepted that the toxicity of both GO and BNNTs is dose- and time-dependent [46,47], and that they do not exert toxicity when used at low concentrations. More importantly, upon coating with a biocompatible polymer like PEG, GO and BNNTs did not show any toxicity against different types of cells even at high concentrations, i.e., up to 100 and 50 mg/L, respectively [48]. Therefore, cell viability data indicate that the functionalization of GO and BNNTs with PEG, a non-toxic andbiocompatible polymer, reduces their cytotoxicity towards human cells.

**Figure 14:**
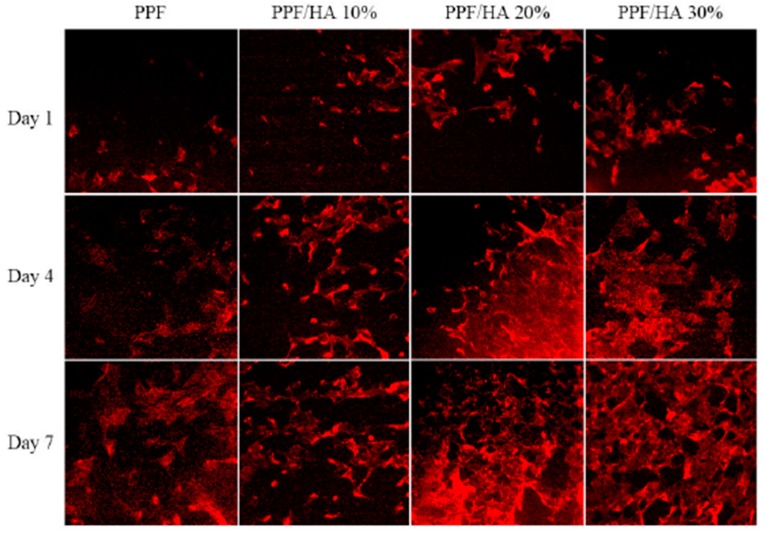
F-actin staining of MC3T3 cells after 1, 4, and 7 days of culture on PPF and PPF/HA nanocomposites with different HA contents. Adapted from [15], with permission from Elsevier.

On the other hand, the proliferation of osteoblast-likeMC3T3 cells on PPF and PPF/HA nanocomposites has been investigated [15], and the results revealed that the cell density gradually increased with increasing HA concentration, by about 37% for the nanocomposite with 30 wt % HA, ascribed to the excellent osteoconductivity and biocompatibility of this inorganic filler. Further, initial cell attachment, cytoskeletal organization and cell spreading were examined at different periods (Figure 14) [15]. After one day, cells grown on the surface of PPF and PPF/HA nanocomposites showed some actin filaments, and more cell-cell interactions were observed on nanocomposites incorporating 20 and 30 wt % HA. After 4 days, networks of actin filaments were found over the cell grown on these nanocomposites, indicating higher complexity of actin filaments as HA composition increases. This actin cytoskeleton organization is a requirement for preserving cell morphology and adhesion between cultured cells and substrate surfaces, as well as for promoting cell spreading and proliferation. Experimental data confirm that PPF-based nanocomposites have excellent in vitro cytocompatibility, therefore are ideal candidates for biomedical applications.

**Figure 15:**
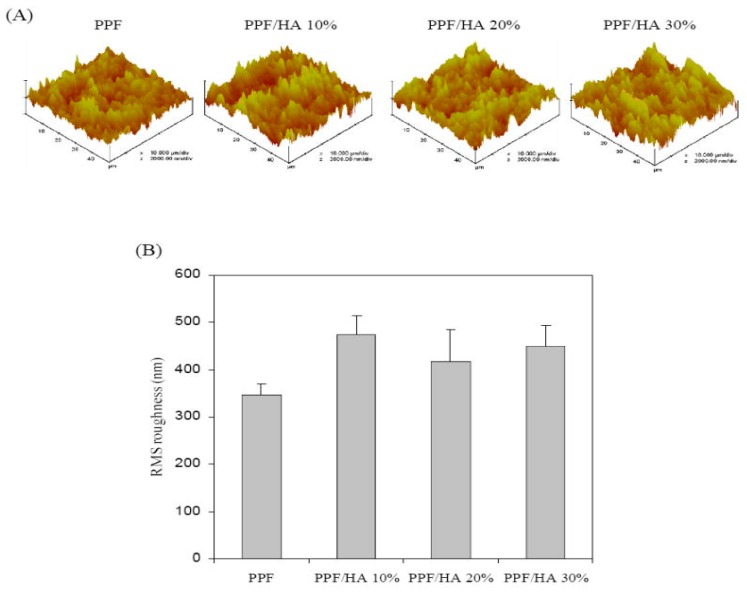
(**A**) AFM images and (**B**) roughness of PPF and PPF/HA nanocomposites with different HA contents. Reprinted from [15], with permission from Elsevier.

An in vitrodegradation study in phosphate buffered saline (PBS) at 37 °C has been carried out to evaluate the biodegradation of PPF/PEG-GO nanocomposites. In general, materials intended for biomedical applications should have a degradation time that matches the regeneration of the tissue they are replacing. PPF exhibited a weight loss of ~3.5% owed to the hydrolytic degradation of the ester linkages that produces fumaric acid and PG as the two main degradation products. An increase in weight loss was detected with increasing nanofiller loading [13], following analogous tendency to the water uptake, and the largest increment (around 60%) was found for the composite with 3.0 wt % PEG-GO. The protein absorption capability of PPF-based nanocomposites was also measured via immersion in a medium containing 10% fetal bovine serum at 37 °C [13,14,15]. The protein concentration was assessed using a Micro BCA protein assay kit. An increase in the protein concentration was detected for PPF/PEG-GO, PPF/PEG-*g*-BNNT and PPF/HA nanocomposites, leading to about 3.5, 2.1 and 1.7-fold increment for the highest nanofiller concentration, respectively. The highest protein adsorption was found for composites with PEG-GO, related to their higher wettability, hence they are expected to be better for promoting cell attachment and proliferation. Further, it is known that the incorporation of nanofillers within a polymer matrix changes the surface topography [49], and consequently affects cell adhesion and growth. PPF/PEG-GO nanocomposites also showed an increase in surface roughness with increasing PEG-GO loading, another factor that is highly advantage for cell proliferation. However, only a moderate increase in roughness was found upon addition of 10 wt % HA nanoparticles to PPF, as shown by the AFM study (Figure 15), and it remained almost constant at higher loadings [15]. In contrast, the degree of hydrophilicity was considerably enhanced upon adding HA, indicating that the cell attachment onto the surface of the nanocomposites was mostly affected by surface wettability rather than by surface topology.

## 5. Conclusions

In this review, the preparation and characterization of PPF-based nanocomposites reinforced with 1D nanofillers (SWCNT, MWCNT and PEG-*g*-BNNTs) as well as 2D nanostructures (GONR, GONP, MSNP, HA and PEG-GO) has been described. The composites have been fabricated by sonication and thermal curing, and their morphology, biodegradation, cytotoxicity, mechanical, thermal, rheological and antibacterial properties have been discussed. The nanocomposites displayed higher biodegradation rate, protein absorption capability, stiffness and strength than PPF. They also kept adequate rigidity and strength under physiological conditions. A clear trend in the reinforcement effect of the nanofillers was found: MSNP > GONP > MWGONR > SWGONR > SWCNT > MWCNT. The higher reinforcing efficiency of the 2D nanofillers compared to 1D ones was attributed to their larger surface area and the presence of surface functional groups that enabled a higher crosslinking density. The nanocomposites exhibited biocide action against human pathogenic bacteria, Gram-positive *S*. *aureus* and *S*. *epidermidis* as well as Gram-negative *P*. *aeruginosa* and *E*. *coli*, though they did not provoke toxicity on human dermal fibroblasts. Composites filled with PEG-modified GO displayed better mechanical, thermal and antibacterial properties as well as higher surface roughness and protein adsorption capability than those reinforced with PEG-*g*-BNNTs or HA, and those filled with surfactant-wrapped SWCNTs exhibited higher stiffness than those incorporating raw SWCNTs, pointing out that the non-covalent functionalization is more effective for improving the performance of PPF. MSNPs led to the highest mechanical improvements, suggesting that the chemical composition of the nanostructures also plays a crucial role on the final nanocomposite properties. These novel biomaterials show huge potential to be employed for tissue engineering applications. They could be used as orthopedic tissues due to their good biocompatibility and tailor-made mechanical properties. They are suitable for cancellous bone defect repair and for the replacement of soft tissues like muscles, tendons, ligaments, fascia, nerves, fibrous tissues, fat, blood vessels, and synovial membranes. However, further research is still required to fully exploit the potential technological applications of these nanocomposites. The development of the optimum formulation for each nanofiller system to meet the specific property requirements is also desirable. Moreover, research is still required to completely guarantee their non-toxicity as well as the environmental safety of their use. In summary, these bionanocomposites appear to have a very bright future for a wide range of applications in the biomedical field.

## Figures and Tables

**Table 1:** Mechanical properties of PPF-based nanocomposites [12,13,14].

Nanofiller (wt %)	*E* (GPa)	σ_y_ (MPa)	Nanofiller (wt %)	*E*_F_ (GPa)	σ_F_ (MPa)
	0.99	39.1	-	0.68	7.5
EG-GO (0.1)	1.36	47.2	SWCNT (0.1)	0.78	14.5
PEG-GO (0.5)	1.72	59.4	MWCNT (0.1)	0.74	18.8
EG-GO (1.0)	2.33	70.3	SWGONR (0.1)	0.74	14.6
EG-GO (2.0)	2.51	74.9	MWGONR (0.1)	0.70	21.5
PEG-GO (3.0)	2.91	90.2	GONP (0.1)	0.84	26.5
PEG-*g*-BNNTs (0.1)	1.14	37.3	MSNP (0.1)	0.90	27.2
PEG-*g*-BNNTs (0.5)	1.29	45.2	SWCNT (0.2)	0.78	11.2
PEG-*g*-BNNTs (1.0)	1.72	56.3	MWCNT (0.2)	0.70	14.5
PEG-*g*-BNNTs (2.0)	1.98	59.5	SWGONR (0.2)	0.79	13.8
PEG-*g*-BNNTs (4.0)	2.34	67.2	MWGONR (0.2)	0.80	18.0
			GONP (0.2)	0.90	27.2
			MSNP (0.2)	0.95	24.8

Young’s modulus *E*, tensile strength σ_y_, flexural modulus *E*_F_, flexural strength σ_F_.
